# Cre recombinase promotes leukemogenesis in the presence of both homozygous and heterozygous *FLT3*-ITD

**DOI:** 10.1038/s41375-024-02259-x

**Published:** 2024-05-08

**Authors:** Min Yang, Zhiyuan Ma, Chonggang Wang, Muammer Cihan Agca, Hongyun Liu, Kezhi Huang, Silke Glage, Regina Rumpel, Alexander Gerbaulet, Axel Roers, Xuemei Liu, Fatih Noyan, Nils von Neuhoff, Arnold Ganser, Ligen Liu, Haiyang Yun, Zhixiong Li

**Affiliations:** 1 Department of Hematology, Hemostasis, Oncology, and Stem Cell Transplantation, Hannover Medical School, Hannover, Germany; 2 Institute for Hematologic Malignancies, East Hospital, Tongji University School of Medicine, Shanghai, China; 3 Institute for Laboratory Animal Science and Central Animal Facility, Hannover Medical School, Hannover, Germany; 4 Institute for Immunology, Medical Faculty Carl Gustav Carus, TU Dresden, Dresden, Germany; 5 Institute for Immunology, University Hospital Heidelberg, Heidelberg, Germany; 6 Department of Gastroenterology, Digestive Disease Hospital, Affiliated Hospital of Zunyi Medical University, Zunyi, China; 7 Department of Gastroenterology, Hepatology, Infectious Diseases and Endocrinology, Hannover Medical School, Hannover, Germany; 8 Department of Pediatric Hematology and Oncology, University Children’s Hospital Essen, University of Duisburg-Essen, Essen, Germany; 9 Department of Hematology, Shanghai Tongren Hospital, Shanghai Jiao Tong University School of Medicine, Shanghai, China; 10 Robert Bosch Center for Tumor Diseases, Stuttgart, Germany; 11 Department of Hematology, East Hospital, Tongji University School of Medicine, Shanghai, China

**Keywords:** Preclinical research, Cancer models

## To the Editor:

Cre recombinase murine models allow the expression of mutated genes in a cell type-specific manner or via an inducible mechanism and has revolutionized biomedical research. However, these models may be associated with some caveats, such as off-target effects and lack of fidelity. In the issue 4 of LEUKEMIA in 2023, Straube et al., described an unexpected observation where Cre expression alone was able to drive early acute myeloid leukemia (AML) in the context of *FLT3*-ITD/ITD (homozygous) [1]. Herein, we found that expression of Cre recombinase induced early AML in the presence of homozygous *FLT3*-ITD in different models, but not in the presence of the *Kit* D814V mutation (murine homolog of human *KIT* D816V mutation). Moreover, Cre recombinase also promoted leukemogenesis in the presence of heterozygous *FLT3*-ITD.

**Fig. 1:**
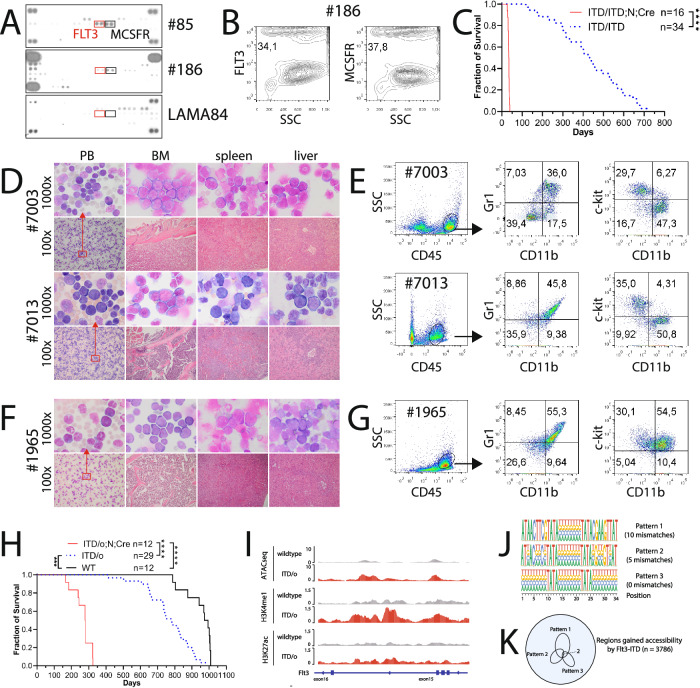
Cre recombinase promoted leukemogenesis in the presence of *FLT3*-ITD. **A** Representative antibody arrays from two patients with AML (#85 and #186). Phosphorylation of FLT3 and MCSFR was observed in patients #85 (FLT3-TKD) and #186 (FLT3-ITD), but not in LAMA84 cells. LAMA84 cells were isolated from a patient with chronic myeloid leukemia in a blast crisis. We did not observe FLT3 or MCSFR phosphorylation in any of the healthy controls analyzed (*n* = 8). Hybridization signals at the corners (three or four) served as positive controls. MCSFR and FGFR2 belong to the most phosphorylated receptor in the AML specimens in our analysis. **B** Representative flow cytometry analyses confirming expression of FLT3 and MCSFR in patient #186. **C** Survival curves of ITD/ITD; *Mcsfr*^*flox*^; *Mxl-Cre*, ITD/ITD; *Fgfr2*^*flox/o*^; *Mxl-Cre*, ITD/ITD; *Mxl-Cre* (3 strains together ITD/ITD;N;*Cre*), and ITD/ITD mice. The animals were not treated with polyinosinic-polycytidylic acid (polyIC), as this was scheduled fo around 6 weeks after birth. No phenotypic differences were observed between TD/ITD; *Mcsfr*^*flox*^; *Mxl-Cre*, ITD/ITD; *Fgfr2*^*flox/o*^; *Mxl-Cre*, and ITD/ITD; *Mxl-Cre*, especially there was no additional acceleration or delay of the disease in TD/ITD; *Mcsfr*^*flox*^; *Mxl-Cre*, and ITD/ITD; *Fgfr2*^*flox/o*^; *Mxl-Cre* mice. ****p < 0.0001. **D** Representative Pappenheim-stained blood smears and cytospins of bone marrow (BM), spleen and liver, and hematoxylin and eosin (H&E)-stained histopathology of BM, spleen, and liver from #7003 (ITD/ITD; *Mcsfr*^*flox*^; *Mxl-Cre*) and #7013 (ITD/ITD; *Mxl-Cre* mice) mice. Note infiltration of myeloblasts in these organs and in the lung (Supplementary Fig. 1A). **E** Flow cytometry analysis of spleen samples from mice #7003 and #7013 demonstrated a population of myeloblast/immature cells with lower side scatter (SSC) and CD45^dim^ expression, which were positive for CD11b, c-Kit, and Gr1, but negative for CD3 and CD19 (Supplemental Fig. 1B). **F** Representative Pappenheim-stained blood smears and cytospins of bone marrow (BM), spleen and liver, and hematoxylin and eosin (H&E)-stained BM, spleen, and liver samples from #1965 (ITD/o; *Fgfr2*^*flox/flox*^; *Mxl-Cre*). **G** Flow cytometry analysis of the spleen sample showing a population of myeloblast/immature cells with lower side scatter (SSC) and CD45^dim^ expression in sample from mouse #1965. These blasts were positive for CD11b, c-Kit, and Gr1, but negative for CD3 and CD19 (data not shown). **H** Survival curves of ITD/o; *Mcsfr*^*flox*^; *Mxl-Cre*, ITD/o; *Fgfr2*^*flox*^; *Mxl-Cre* (2 strains together ITD/o;N;*Cre)*, ITD/o, and wildtype (WT) mice. One mouse from ITD/o; *Fgfr2*^*flox*^; *Mxl-Cre* was treated with polyIC. ***p < 0.001, ****p < 0.0001. **I** Illustration of chromatin profiling on accessibility (ATACseq) and modification states (ChIPseq on H3K4me1 and H3K27ac) in wildtype vs. ITD/o mouse HSPCs. An *FLT3*-ITD-associated enhancer was identified in the intron 15 of *Flt3* gene in ITD/o cells, marked by a high enrichment of H3K4me1, and modest levels of ATACseq and H3K27ac. Interesting, Straube et al. reported the presence of a neomycin resistance cassette (NRC) flanked by loxP sites in intron 15 of the *Flt3* gene in *FLT3*-ITD mice, and the excision of NRC in the presence of Cre [1]. **J** Visualization of motif logos representing pseudo loxP sites using three parameters. **K** Identification of chromatin regions containing pseudo loxP sites in the context of accessibility gained by *FLT3*-ITD.

To identify cooperating partners for the *FLT3*-ITD in the development of AML, we analyzed the activation of ≥ 42 receptor tyrosine kinases in primary samples from AML patients using a phospho-kinase antibody array. The phosphorylated kinases, Macrophage colony stimulating factor receptor (MCSFR) and Fibroblast growth factor receptor 2 (FGFR2) were detected in 78% and 31% of AML patients (*n* = 90), respectively (Fig. 1A and data not shown). The expression of MCSFR on blasts was confirmed in all analyzed primary samples with phosphorylated kinases (*n* = 32) by flow cytometric analysis (Fig. 1B). In a separate cohort, we detected MCSFR expression almost in all primary samples from AML patients (*n* = 125) by flow cytometric analysis (data not shown). Importantly, MCSFR was identified as a therapeutic target in AML leukemia [2]. Moreover, MCSFR is crucial for leukemic stem cells (LSC) potential induced by the MOZ-TIF2 fusion [3]. Interestingly, FGFR2 has been suggested to be important for leukemic-regenerating cells (LRCs) that are induced by chemotherapy and responsible for disease relapse [4].

To test whether MCSFR or FGFR2 is important for LSC potential induced by *FLT3*-ITD, we crossed *FLT3*-ITD knock-in mice with *Mcsfr*^*flox*^ and *Mxl-Cre, and Fgfr2*^*flox*^ and *Mxl-Cre*, to generate ITD/ITD; *Mcsfr*^*flox*^; *Mxl-Cre* (homozygous *FLT3*-ITD) and ITD/ITD; *Fgfr2*^*flox*^; *Mxl-Cre* mice. ITD/ITD; *Mxl-Cre* mice were used as a control. To our surprise, the ITD/ITD; *Mcsfr*^*flox*^*; Mxl-Cre*, ITD/ITD; *Fgfr2*^*flox/o*^; *Mxl-Cre* and ITD/ITD; *Mxl-Cre* mice developed an early aggressive AML approximately 34 days after birth (*n* = 16, Table 1, Fig. 1C–E), whereas the median survival of the ITD/ITD mice was 431 days (*p* < 0.0001). The animals demonstrated a very high degree of leukocytosis (white blood cell (WBC): 477.9 ± 181.2/µl, *n* = 14 vs. control mice: 5.8 ± 1.4/µl, *n* = 7; Fig. 1D). Such high leukocytosis with WBC > 400,000/µl was not previously observed in any of our murine models including >200 animals with acute leukemia [5, 6]. Diseased mice had pronounced splenomegaly (705 ± 215 mg, *n* = 15 vs. control: 179 ± 21 mg, *n* = 7) and hepatomegaly was observed in the majority of diseased mice (1783 ± 550 mg, *n* = 15 vs control: 1262 ± 230 mg, *n* = 7). In contrast, *Kit* D814V mutation, murine homolog of *KIT* D816V, which is a very common mutation found in patients with systemic mastocytosis and in some AML patients, did not cooperate with Cre to induce early AML (Table 1).

**Table 1:** Development of early AML.

Strains	Number of diseased mice (*n*)	Latency (days)
ITD/ITD; *Mcsfr*^*flox*^; *Mxl-Cre* ITD/ITD; *Fgfr2*^*flox/o*^; *Mxl-Cre*	12	34
ITD/ITD; *Mxl-Cre*	4	34
*total*	16	34
*Kit* D814V^flox^; *Mxl- Cre* [11]	0 (out of 35 analyzed mice)	n.a.

*Mcsfr*^*flox*^ heterozygote and homozygote, *Fgfr2*^*flox/o*^ heterozygote, *n.a.* not applicable.

Although ITD/o; *Mcsfr*^*flox*^*; Mxl-Cre* and ITD/o; *Fgfr2*^*flox*^*; Mxl-Cre* (ITD/o = heterozygous *FLT3*-ITD) mice did not develop early AML, a diagnosis of AML was made in all analyzed mice (*n* = 12) at the endpoint analysis (Fig. 1F, G). Moreover, these mice had much shorter survival than mice with ITD/o alone (279 vs. 783 days, *p* < 0.0001, Fig. 1H). A similar observation with shorter survival for mice carrying ITD/o and Cre was also made by others (personal communications by Florian H. Heidel) [7]. In another study, all mice (*n* = 9) transplanted with bone marrow (BM) cells from ITD/o; *Fgfr2*^*flox/flox*^*; Mxl-Cre* or ITD/o; *Mxl-Cre* mice developed AML around 7 months after transplantation, while only chronic myelomonocytic leukemia (CMML) was observed in diseased mice (*n* = 3) transplanted with ITD/o BM cells approximately 14 months after transplantation (Supplementary Fig. 2). Importantly, the median survival of mice transplanted with ITD/ITD (*n* = 3), ITD/ITD; *p53*^+/−^ [6], and ITD/ITD; *p53*^−/−^ [6] BM cells was around 6, 5, and 5 months, respectively. Taken together, Cre recombinase promotes leukemogenesis of both homozygous and heterozygous *FLT3*-ITD.

At the molecular level, chromatin profiling revealed the existence of a poised enhancer in intron 15 of *Flt3* in *FLT3*-ITD/o mouse hematopoietic stem/progenitor cells but not in wildtype counterparts, which were marked by increased chromatin accessibility, enrichment of H3K4me1, and lower levels of H3K27ac (Fig. 1I). Cre expressions resulted in a fully active enhancer mapping at intron 15 of *Flt3* and increased the expression of *Flt3* gene (Supplementary Fig. 3A, B), suggesting that Cre-mediated recombination may facilitate chromatin activation. Recently, we described that the *FLT3*-ITD mutation alone can remodel the chromatin landscape to prime the development of full-blown leukemia in cooperation with other mutations [8]. A possible causative mechanism may involve Cre cleavage of genomic sites activated by *FLT3*-ITD. We focused on 3786 genomic regions that gained chromatin accessibility in the presence of *FLT3*-ITD [8], and then scanned for three loxP motif patterns to identify pseudo loxP sites [9] (Fig. 1J). Interestingly, we identified two pseudo-loxP sites mapping to the *FLT3*-ITD open chromatin region (Fig. 1K). Whether Cre enhances leukemogenesis of *FLT3*-ITD through these pseudo-loxP sites needs to be determined. In ongoing studies we wish to understand the underlying molecular mechanism for the development of AML by Cre and FLT3-ITD in more details.

In summary, our data not only confirm the cooperation between Cre and *FLT3*-ITD homozygous in the induction of AML described by Straube et al., but also provide the first evidence of enhanced leukemogenesis of *FLT3*-ITD heterozygous by Cre. *FLT3*-ITD knock-in mice have been used to identify cooperative partners, also in the presence of Cre [10]. Our data strongly support the findings of Straube et al. and indicate the need for a careful study design and interpretation of the data when using the Cre-loxP recombination system. In our hands, ITD/ITD in the presence of Cre activity does not allow to investigate the role of *MCSFR* or *FGFR2* in the pathogenesis of *FLT3*-ITD. Whether ITD/o in the presence of Cre is suitable for testing of cooperative events remains to be determined.

### Supplementary information

Suppl. data
