# Supplementary material for: Cre recombinase promotes leukemogenesis in the presence of both homozygous and heterozygous FLT3-ITD
Source: Leukemia. 2024 May 8;38(6):1437–9. doi: 10.1038/s41375-024-02259-x (PMC11147757; doi:10.1038/s41375-024-02259-x)
Supplement: Supplementary file 1 — Suppl. data [file 41375_2024_2259_MOESM1_ESM.docx]

**Supplementary data (Yang M et al. 2024)**

**SUPPLEMENTARY METHODS**

**Transgenic mice**

We obtained *FLT3*-ITD knock-in, *Mcsfr^flox^*, *Mxl-Cre, and Fgfr2^flox^* mice from Jackson Laboratory (Bar Harbor, ME). *FLT3*-ITD knock-in mice with C57BL/6J background that carry the human internal tandem duplication (ITD) mutation W51 in exon 14 of the endogenous murine *Flt3* locus were generated in Gilliland’s laboratory [1]. *Kit* D814V^flox^; *Mxl- Cre* were generated as previously described [2]. *FLT3*-ITD knock-in mice were crossed with *Mcsfr^flox^* and *Mxl-Cre, and Fgfr2^flox^*  and *Mxl-Cre,* to generate ITD/ITD; *Mcsfr^flox^*; *Mxl-Cre* (homozygous *FLT3*-ITD) and ITD/ITD; *Fgfr2^flox^*; *Mxl-Cre* mice. We used ITD/ITD; *Mxl-Cre* mice as a control.

**Mouse monitoring and tumor phenotyping [3]**

Animal experiments were approved by the local ethical committee LAVES (Niedersächsisches Landesamt für Verbraucherschutz und Lebensmittelsicherheit) and were performed according to the respective guidelines. All mice were kept in the animal houses of Hannover Medical School. Mice were killed for necropsy when the experiment termination criteria were met or the endpoints of the experiment were reached [4]. Tumor phenotyping was performed as previously described.

**Genomic DNA isolation and genotyping [3]**

Genomic DNA was generally isolated from either ear biopsy using the DNA Blood Mini Kit. For the genotyping analysis of FLT3-ITD and wild-type FLT3, *Mcsfr^flox^*, *Mxl-Cre, and Fgfr2^flox^ ,* primers and PCR protocols from the Jackson Laboratory were used. The PCR products were subjected to agarose gel electrophoresis.

**Bone marrow transplantation and polyIC treatment**

Bone marrow cells were isolated from ITD/o; *Fgfr2^flox/flox^; Mxl-Cre*, ITD/o; *Mxl-Cre*, ITD/o, or ITD/ITD mice and were transplanted into lethally irradiated mice. Around 6 weeks after transplantation, recipients carrying *Mxl-Cre* were treated with polyinosinic-polycytidylic acid (polyIC), approximately 3 times intraperitoneal injections, 200 - 500 µg each, volume 10 ml/kg, every two days) to induce Cre recombinase expression [2].

**Immunophenotypic analysis [3]**

Generally, 1×10^6^ cells from specimens of each organ were washed with PBS and suspended in 100μl of FACS buffer and then were stained with antibodies for 30 min away from light at 4°C. Leukemic cells were stained at least with immature myeloid markers (c-Kit and CD34), mature myeloid markers (Gr-1, CD11b), an erythrocyte marker (TER119), and lymphocytic markers (CD3, CD4, CD8, and CD19). After incubation, cells were washed with PBS and resuspended in 400μl of FACS buffer for acquisition by FACS flow cytometer. The measurement of cell surface markers was performed using the BD FACS Canto or BD FACS Canto II system.

**Antibody arrays and patient samples [3]**

Studies with human samples were approved by the ethical committee of the Hannover Medical School. Human RTK antibody arrays were performed according to the manufacturer’s protocol, but with 2 mg protein lysate per array [5]. We studied tumor specimens from patients with AML who had been diagnosed at the Hannover Medical School. Blood and/or bone marrow (BM) samples from patients with AML were collected at diagnosis after informed consent. Mononuclear cells from all samples studied were immediately isolated by centrifugation over Ficoll gradient and were freshly used or stored at –180^0^C until further use.

**Enhancer identification and analysis of Flt3 expression [6]**

We analyzed the ATACseq and ChIPseq data generated in our previous study [6] for the characterization of the enhancer sequence in the intron 15 of the *Flt3* gene. Normalized data tracks in Bigwig format were downloaded from GSE146616 (ATACseq) and GSE146663 (ChIPseq). They were uploaded to Integrative Genomics Viewer (IGV) for visualization of the chromatin states at the indicated genomic locus. Expression levels of the *Flt3* gene were determined by RNA-seq. For each genotype, normalized reads of all exons of *Flt3* gene represented by Reads Per Kilobase per Million mapped reads (RPKM) were extracted and plotted. Two replicates of each genotype were utilized for the analysis.

**Data analysis and statistics [3]**

The FACS data were analyzed using FlowJo software (Tree Star, OR, USA). Statistical analyses were generated with Graphpad Prism 6 or 7 (San Diego, CA). The results were represented as means ± SD. Significant differences in Kaplan-Meier survival curves were evaluated using the log-rank test. P-values less than 0.05 were considered statistically significant.

**SUPPLEMENTARY FIGURES**


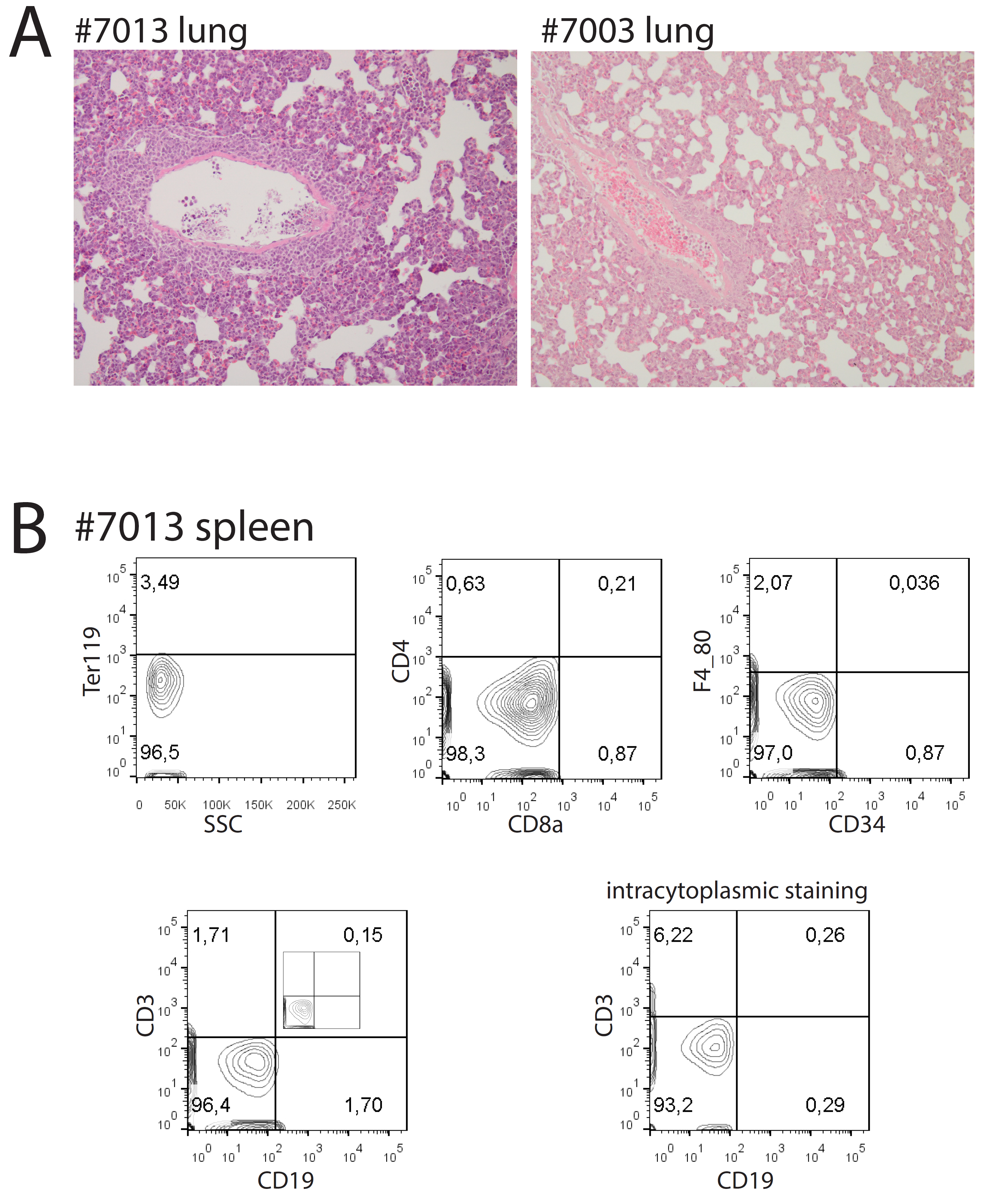


**Supplementary Fig. 1 A** Histology showing the strong infiltration of blasts in the lung of mice #7013 and #7003. **B** Representative immunophenotypic analyses of diseased mice. No expression of Ter119, CD4, CD8 F4/80, CD34, Cd3, CD19, or cyCD3 was observed on the surface of myeloblasts from mouse #7013.


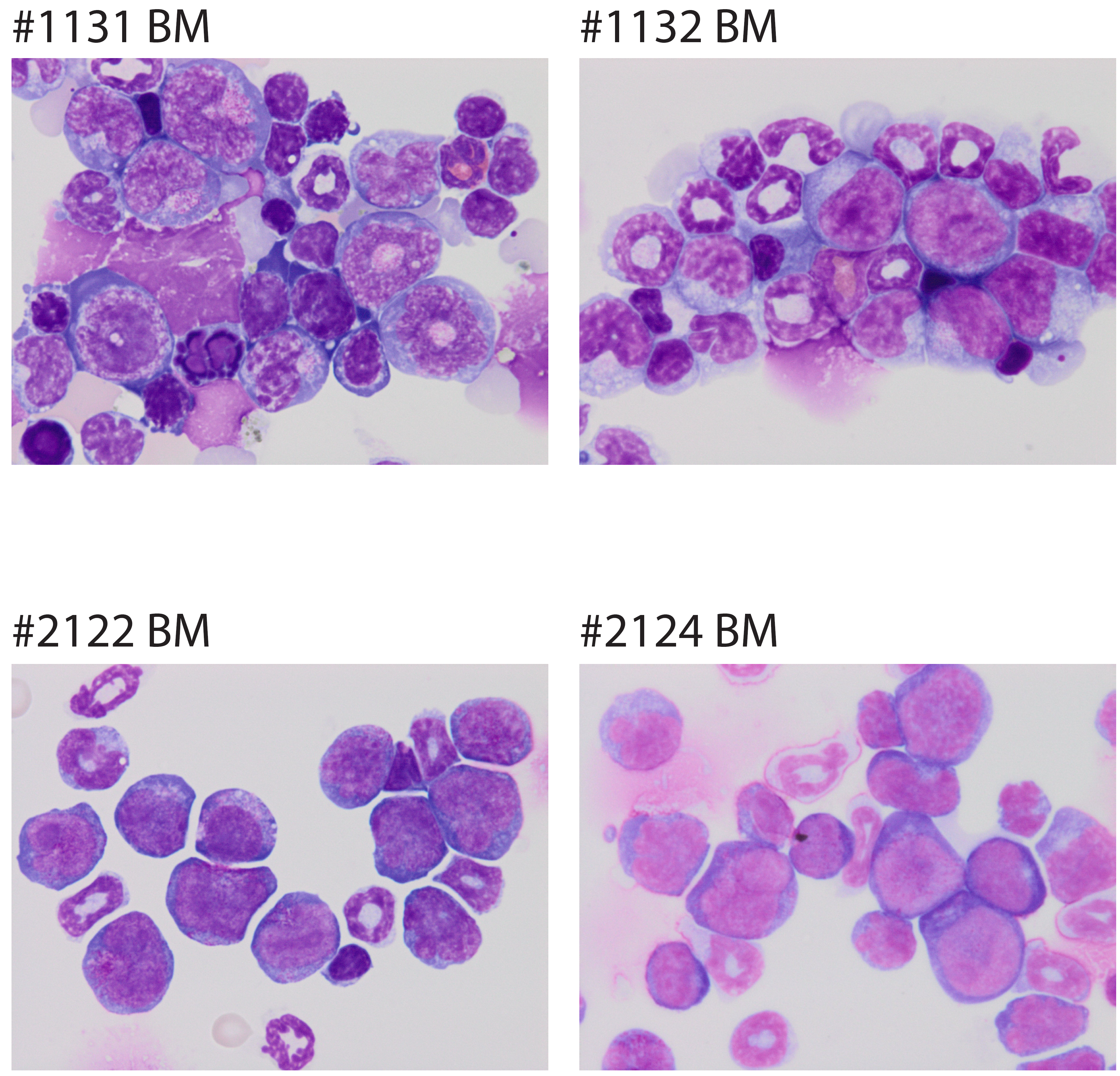


**Supplementary Fig. 2** Representative Pappenheim-stained cytospins of bone marrow from mice #1131, #1132, #2122, and #2124. Mice #1131 and #1132 were transplanted with ITD/o bone marrow cells developed a chronic myelomonocytic leukemia-like disease, while strong myeloblasts infiltration of mouse #2122 transplanted with ITD/o; *Fgfr2^flox/flox^; Mxl-Cre* cells and mouse #2124 mice with ITD/o; *Mxl-Cre* cells.

**Supplementary Fig. 3 A** Illustration of chromatin profiling on accessibility (ATACseq) and enhancer states (H3K4me1 and H3K27ac) in mouse HSPCs of *Npm1*^flox/o^; Mx1-Cre without vs. with ITD/o. **B** Quantification of Flt3 mRNA expression measured by RNA-seq using Reads Per Kilobase per Million mapped reads (RPKM) in wildtype and mutant mouse HSPCs.

**References**

1. Lee BH, Tothova Z, Levine RL, Anderson K, Buza-Vidas N, Cullen DE, et al. . FLT3 mutations confer enhanced proliferation and survival properties to multipotent progenitors in a murine model of chronic myelomonocytic leukemia. Cancer Cell. 2007;12:367-380.

2. Gerbaulet A, Wickenhauser C, Scholten J, Peschke K, Drube S, Horny HP, et al. . Mast cell hyperplasia, B-cell malignancy, and intestinal inflammation in mice with conditional expression of a constitutively active kit. Blood. 2011;117:2012-2021.

3. Yang M, Pan Z, Huang K, Busche G, Liu H, Gohring G, et al. . A unique role of p53 haploinsufficiency or loss in the development of acute myeloid leukemia with FLT3-ITD mutation. Leukemia. 2022;36:675-686.

4. Yang M, Huang K, Busche G, Ganser A, Li Z. Activation of TRKB receptor in murine hematopoietic stem/progenitor cells induced mastocytosis. Blood. 2014;124:1196-1197.

5. Stommel JM, Kimmelman AC, Ying H, Nabioullin R, Ponugoti AH, Wiedemeyer R, et al. . Coactivation of receptor tyrosine kinases affects the response of tumor cells to targeted therapies. Science. 2007;318:287-290.

6. Yun H, Narayan N, Vohra S, Giotopoulos G, Mupo A, Madrigal P, et al. . Mutational synergy during leukemia induction remodels chromatin accessibility, histone modifications and three-dimensional DNA topology to alter gene expression. Nat Genet. 2021;53:1443-1455.
